# Effects of Railway Elevation, Operation of a New Station, and Earthquakes on Railway Noise Annoyance in Kumamoto, Japan

**DOI:** 10.3390/ijerph15071417

**Published:** 2018-07-05

**Authors:** Yasuhiro Murakami, Takashi Yano, Makoto Morinaga, Shigenori Yokoshima

**Affiliations:** 1 Department of Architecture, Sojo University, Ikeda 4-22-1, Nishi-ku, Kumamoto 860-0082, Japan; yasuhiro@arch.sojo-u.ac.jp; 2 Kumamoto University, Kurokami 2-39-1, Chuo-ku, Kumamoto 860-8555, Japan; 3 Defense Facilities Environment Improvement Association, Shiba 3-41-8, Minato-ku, Tokyo 105-0014, Japan; morinaga@dfeia.or.jp; 4 Kanagawa Environmental Research Center, Shinomiya 1-3-39, Hiratsuka 254-0014, Japan; yokoshima.7c7q@pref.kanagawa.jp

**Keywords:** annoyance, railway noise, railway elevation, new station, Kumamoto earthquake, logistic regression analysis

## Abstract

This study investigated the effects of railway elevation, operation of a new station, and earthquakes on railway noise annoyance in two areas along a conventional railway line (CRL) adjacent to the Kyushu Shinkansen line: the north area with the CRL elevation and the south area with the operation of the new station, both of which occurred in March 2016. In April 2016, Kumamoto region was struck by a series of large earthquakes, prompting their inclusion in this study, as frequent aftershocks with loud ground rumbling might make people more sensitive to railway noise and vibration. Socioacoustic surveys were performed in both areas before and after the earthquakes. Because very few respondents in the north area reported that they were “highly annoyed,” further analysis was conducted on data from the south area. The exposure–annoyance relationship was found to be significantly higher in 2017 than in 2011 despite lower noise exposure. Multiple logistic regression analysis showed that *L*_den_, noise sensitivity, and serious damage by the earthquakes in addition to the operation of the new station significantly affected the annoyance in both detached and apartment houses. However, when the earthquakes caused minimal damage, they did not significantly affect annoyance.

## 1. Introduction

**Figure 1:**
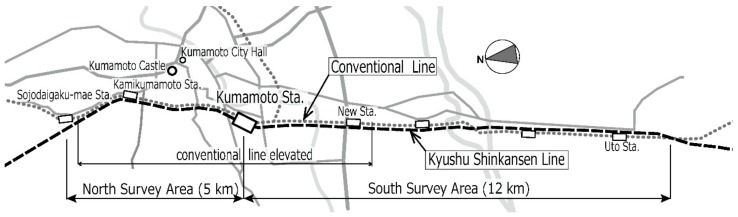
Survey area.

**Figure 2:**
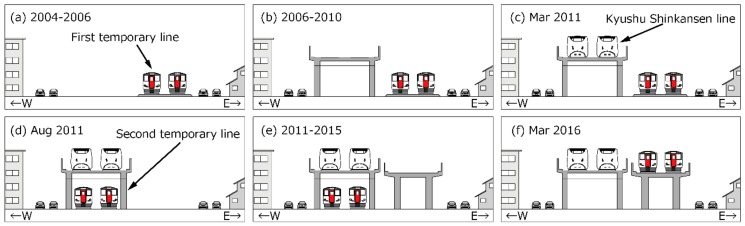
Timeline of railway construction in the north area. (**a**) Conventional railway moved to the 1st temporary line; (**b**) Construction of the elevated KSL; (**c**) Operation of KSL; (**d**) Conventional railway moved to the 2nd temporary line; (**e**) Construction of the elevated conventional railway line; (**f**) Operation of the elevated conventional railway line.

The Shinkansen (super express) railway network has been developed throughout Japan since the Tokaido Shinkansen line started operating in 1964. As part of the development of the Shinkansen network, the Kyushu Shinkansen line (KSL) that operates from Fukuoka to Kagoshima via Kumamoto, which is the midpoint between the two cities, was opened in 2011. The conventional railway line (CRL) and KSL are adjacent in an area that stretches 5 km to the north of Kumamoto station (north area) and in another area that stretches 12 km to the south of the station (south area), as shown in Figure 1. After the opening of the KSL, a second temporary CRL (see Figure 2d) was opened in August 2011. Then, the CRL was elevated in March 2016 in the north area, and a new station was opened in March 2016 in the south area. Such situations caused step changes in railway noise exposure in the areas along the railway lines; the railway elevation with noise barriers reduces noise exposure near the barriers by diffraction, and the operation of the new station also reduces noise emissions as trains slow down as they approach/depart from the station. Tetsuya et al. [1] conducted socioacoustic surveys before and after the commencement of KSL operations. They reported that noise annoyance decreased slightly despite an increase in railway noise exposure, following the opening of the KSL. Large earthquakes with intensities of 7 (the highest intensity) on the Japanese seven-stage seismic scale unexpectedly struck the Kumamoto region twice, on 14 and 16 April 2016. Such serious disasters could affect community response to noise [2,3]. Therefore, the objective of this study was to investigate the effects of the elevation of the CRL in March 2016, the operation of a new station in March 2016, and the occurrence of earthquakes in April 2016 on railway noise annoyance, based on surveys conducted before and after the interventions/events.

The primary interest in step–change (or intervention) studies is to assess whether people overreact to changes in noise exposure compared with previous steady–state conditions. Brown and van Kamp defined the effects caused by noise exposure under steady–state conditions as “exposure effects” and the additional effects due to noise exposure change as “change effects” [4,5]. In relation to change effects, they defined the cases in which increased or decreased responses were proportional to a change in noise exposure as an “excess response”, and those in which increased or decreased responses were inversely proportional to a change, as an “under response”. The use of these terms has been adopted in this paper.

Brown and van Kamp systematically reviewed 42 step–change studies, and for changes in road traffic noise at the source, they reported an excess response in comparison with steady–state exposure–response relationships [4]. They called a change in noise at the source “Type 1” and that during noise propagation as “Type 2.” Thus, the change in noise associated with railway elevation is considered Type 2 and that related to the operation of a new station is considered Type 1. Recently, Brown and van Kamp expanded their review to include studies from 1980–2014 [6]. They listed 11 explanations interpreting the excess response and identified three of them as plausible explanations: changes in the modifiers of exposure–response relationships, differential response criteria, and retention of coping strategies [5]. However, of all the studies they reviewed, only six were related to railway noise [4].

For example, Ohrstrom showed the effectiveness of different countermeasures for reducing vibration annoyance and noise annoyance in a socioacoustic survey in Kungsbacka, Sweden [7]. A pilot study on the effects of rail grinding in relation to railway noise annoyance was performed by Moehler et al., who indicated considerable reduction in noise exposure and significant decrease in annoyance [8]. Schreckenberg et al. conducted two socioacoustic surveys on railway noise annoyance in Germany: one before and after railway expansion, and the other before the opening of a new line [9]. They emphasized that offering affected residents complete and timely information might prevent mistrust and fears that strongly determine levels of annoyance. Liepert et al. and Schreckenberg et al. both conducted socioacoustic surveys before and after rail grinding in two areas in South Germany: one in which information on rail grinding effects was provided and the other in which it was not [10,11]. They reported that noise annoyance decreased significantly after rail grinding in the area in which information was offered, suggesting that providing information might positively affect the response to railway noise change. In performing three surveys before and after commencement of operations on a new railway line in Hong Kong, Lam et al. reported that noise annoyance decreased, even though noise exposure increased slightly [12]. Their findings suggested the possibility of adaptation to a new noise source and accentuated the impact of media reporting about the railway line. Overall, these studies appeared to show no clear evidence of change effects.

Though many intervention studies regarding railway noise and noise caused by other forms of transportation have been published, only one intervention study exists in Japan [13], and to date, no study has investigated the effect of earthquakes on noise annoyance anywhere in the world. Therefore, this study is unique as it considers the effects of noise caused by earthquakes in this context and also contributes to an improved understanding of noise impact assessment requirements for railway infrastructure construction and railway noise policy in Japan.

## 2. Methods

### 2.1. Context of the Study

The social surveys were conducted in the areas ranging from Kumamoto station to Sojodaigaku-mae station (north area), and from Kumamoto station to Uto station (south area), where the KSL and CRL are adjacent, as shown in Figure 1. The target houses were detached and apartment houses located within 150 m of the railway. In the north area, the KSL and CRL elevations had been constructed over a period of more than 10 years. In March 2011, the KSL commenced operation and the CRL was moved under the KSL in August 2011. Subsequently, the elevated CRL was constructed along the route of the original CRL, and it began operation in March 2016. In the south area, although a new station was operated in March 2016, the overall condition did not change after the commencement of operation of the KSL. Such interventions caused step changes, usually resulting in reductions in railway noise exposure linked to the propagation of noise from the elevated railway and to the slower train speeds near the new station. Large earthquakes unexpectedly struck and damaged the Kumamoto region in 2016. Table 1 summarizes the survey plans and remarkable events such as the operation of the KSL and the occurrence of the Kumamoto earthquakes. This study compared the annoyance responses of Surveys III (2012) and IV (2016) in the north area and Surveys II (2011) and V (2017) in the south area. There were approximately 3500 houses in the north area and 3800 in the south area. These houses were assigned uniformly to Surveys I–V. This study constitutes a cross–sectional study rather than a longitudinal study because the response rates were rather low (as shown in Section 3.1); a longitudinal study based on repeated interviews with the same respondents was unrealistic.

All the surveys used almost identical question items and annoyance scales. The common question items were classified under the following headings: housing, residential environment, annoyance caused by environmental pollutants, activity interference because of railway operation, and personal factors. In Surveys IV and V, additional questions relating to the earthquakes were added. The questionnaires were titled as “Survey on living environment”. The questionnaires were numbered and identified the respondents by address rather than individual names. They were distributed by hand together with the request letters explaining the intent of the study. Only one questionnaire was provided for a single adult (18 years old or above) per household, and the completed ones were returned by mail. The respondents were selected by the family members themselves depending on the member with the closest birthday, i.e., the person with his/her birthday nearest to the date mentioned in the request letter.

Conventional railway noise, Shinkansen noise, and total noise annoyances were evaluated using the 5-point verbal (“not at all”, “slightly”, “moderately”, “very” and “extremely”) and 11-point numerical (extremes labeled “not at all” and “extremely”) scales proposed by the International Commission on Biological Effects of Noise [14]. Noise annoyance due to general construction activities, that is, those not specifying the elevation and the new station, and the vibration disturbances caused by trains passing by, was also evaluated using the ICBEN 5-point verbal scale. However, any effects of lighting due to the trains and the railway lines were not evaluated. The questions pertaining to annoyance, which used for an 11-point numerical scale, are listed in the Appendix A. Responses to any of the top three categories from the 11-point numerical scale were classified as “highly annoyed.” In Survey II, which was conducted during August–September 2011, the respondents were requested to evaluate the level of annoyance over the previous month because the KSL had commenced operation in March 2011. In the other surveys, the respondents were requested to evaluate the level of annoyance over the previous 12 months.

These surveys were not approved by an ethical committee, but informed consent was taken from the respondents by explaining that they would not be identified, and that the data collected during the survey would be used for academic purposes only.

### 2.2. Characteristics of the Respondents

Table 2 summarizes the number of respondents, response rates, and distributions of sexes, their ages, number of family members, and ownership of houses in Surveys II–V. The total number of respondents was approximately 330–400. The response rate of individuals living in detached houses in the north area, which is an older residential area, was 46–61%, i.e., higher than that in the south area (33–39%). However, the response rate of individuals living in apartment houses was low in both areas (15–25%). The low response rate from individuals living in apartment houses is quite expected in Japan because younger people (who tend to live in apartment houses) are often busy with work and children. Hence, they seem unwilling to answer questionnaire surveys. Males and females provided about 40% and 60% of the responses, respectively. In detached houses, older people in their 60s and 70s or above represented 60–70% of respondents in the north area and 50–60% in the south area. The proportion of younger people living in apartment houses was greater than those in detached houses. Overall, the distributions of sex and age were reasonably uniform among the four surveys. Across all the surveys, only 3–5% of the respondents living in apartment houses had more than five family members but more than 10% of respondents living in detached houses in the south area had more than five family members. The rates of owned detached houses were 75–93% in all the surveys, whereas the rates of owned apartment houses in Surveys II, III, VI, and V were 70, 32, 46, and 18%, respectively. The difference in the rates of owned apartment houses in the north area between 2012 and 2016 was 14%, whereas that in the south area between 2011 and 2017 was 52%.

### 2.3. Noise and Vibration Measurements

Considering the railway conditions and the Shinkansen train speed in the north and south areas, noise measurements were conducted at one side of the railway at one point in the north area in 2012 and at two points in the south area in 2011, both sides of the railway at one point in the north area in 2016, and at three points in the south area in 2017. Sound level meters (RION NL–21 and NL–22, IEC 61672­–1, Class 2) with all—weather wind screens were placed 12.5 m from the center of the nearest track (reference points) at a height of 1.2 m above the ground, and A—weighted sound pressure level with a 1 s time constant was measured every 0.1 s for 24 h. Measurements at points 12.5 m from the track were simultaneously acquired, and separate short–term noise measurements were also performed at 3–4 points within 100 m of the track at 6 sites in the north area in 2012, 7 sites in the south area in 2011, 5 sites in the north area in 2016, and 6 sites in the south area in 2017. These measurements represented a maximum of 20 events each for conventional and Shinkansen train. The energy–averaged *L*_AE_ values of the highest 10 events were calculated as representative noise levels for both trains. The 24 h noise indices *L*_Aeq,24h_ and *L*_den_ were obtained based on the *L*_A*E*_ values and the number of trains from the railway timetable. The horizontal distance reduction equation for each site was formulated using logarithmic regression with *L*_A*E*_ values for railway noise at the various points. The vertical propagation was formulated using three–order regression with vertical measurements at five apartment houses located 10–150 m from the railway line.

The vibration level was measured on the ground at the same points as those used for the noise measurements, by a vibration level meter (RION VM–53A) with a time constant of 0.63 s and frequency weighting (Table A1) as shown in Appendix B [15]. *L*_Vmax_, the arithmetic average of the maximum vertical acceleration levels of the upper 10 vibration events, was obtained for each house using the horizontal distance reduction equation as well as the noise level. Moreover, the vibration level corresponds to the outdoor (no indoor) level. While the vibration level for a detached house does not reduce to a great extent indoors, that for an apartment house is generally so low that the residents may hardly perceive it.

### 2.4. Analysis

To investigate the effects of the step change in railway noise exposure due to the interventions, multiple logistic regression analysis was conducted. After the CRL elevation in the north area and the operation of the new station in the south area, the Kumamoto region was unexpectedly struck by the large earthquakes, which could have possibly affected the respondents’ reactions to noise and vibration from the railways. The degree of damage caused by the earthquakes was used as the intervention factor instead of the survey year. In Surveys IV and V, the degree of damage due to the earthquakes was ascertained based on the following criteria: (1) not at all damaged; (2) furniture and wares partially damaged; (3) house partially damaged; (4) house half damaged; and (5) house fully damaged. The first two and final three criteria were categorized as “minimal” and “serious” damage, respectively. The degree of damage before the earthquakes was classified as “no damage.” Thus, relative to “no damage”, “minimal” was considered to be the combined effect of both the step change and the minor damage due to the earthquakes on annoyance, and “serious” was considered as the combined effect of the step change and the major damage.

The dependent variable was “highly annoyed”, and the independent variables were *L*_den_, sex, age, sensitivity to noise, use of trains, frequency of opening living room windows in the season, and damage due to the earthquakes. Noise and vibration propagation could differ in relation to both detached and apartment houses; therefore, their effects on the residents of each housing type might also be different. Consequently, multiple logistic regression analysis was conducted separately for the detached houses and apartments. As the social surveys were conducted in summer 2012 and in winter 2016 in the north area and in the summers of 2011 and 2017 in the south area, the frequency of opening windows might have been different between surveys. Sensitivity to noise was evaluated using a 5-point verbal scale (“not at all”, “slightly”, “moderately”, “very” and “extremely”), and the first three and second two criteria were categorized as “not sensitive” and “sensitive,” respectively. The use of trains was evaluated using a 5-point verbal scale (“frequently”, “sometimes”, “no preference”, “seldom” and “not at all”), and the first three and second two criteria were categorized as “use” and “not use”, respectively. The opening of living room windows was evaluated using a 4-point verbal scale (“not at all/seldom”, “sometimes”, “often” and “always”), and the first two and second two criteria were categorized as “close” and “open”, respectively. All the statistical analyses were performed using JMP 11 (SAS Institute Inc., Tokyo, Japan).

## 3. Results

### 3.1. Number of Passing Trains

Table 3 compares the number of passing trains during daytime, evening, and nighttime between Survey III (2012) and Survey IV (2016) in the north area and between Survey II (2011) and Survey V (2017) in the south area. The number of conventional passenger cars, Shinkansen trains, and freight trains were about 80, 130, and 10, respectively, in the north area and about 140, 130, and 10, respectively, in the south area. The number of conventional passenger cars was fewer in the north than in the south, through which another CRL passes. In the north area, the number of passenger cars and freight trains were almost the same before and after the track elevation. The total number of Shinkansen trains decreased because of the train schedule after the track elevation in the north area. In the south area, the number of passenger cars and Shinkansen trains increased during the daytime.

### 3.2. Noise and Vibration Exposure

The average noise exposure levels and standard deviations at the houses of the respondents in each survey are summarized in Table 4. The conventional railway noise exposure largely decreased from 2012 to 2016 in the north area and from 2011 to 2017 in the south area because of the railway elevation and the operation of the new station, respectively. For example, *L*_den_ of the conventional railway decreased by 8 dB (from 49 to 41 dB) in the north area and by 6 dB (from 49 to 43 dB) in the south area. However, Shinkansen noise exposure did not change between 2012 and 2016 in the north area or between 2011 and 2017 in the south area because the situation of the Shinkansen railway and the traffic volume remained largely unchanged.

**Figure 3:**
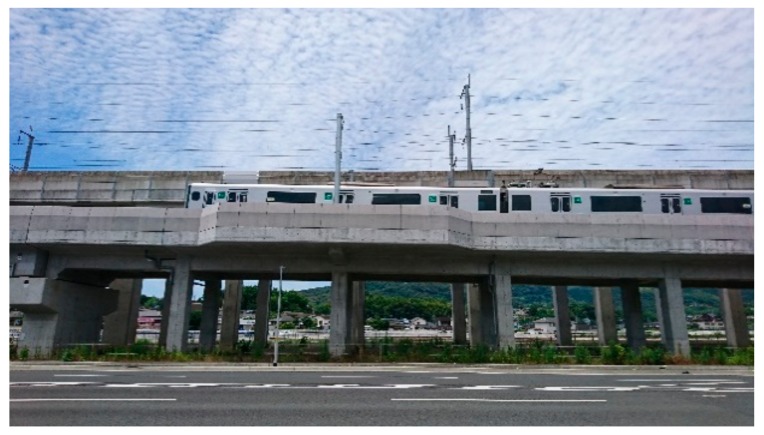
A train passing on the forward CRL (the elevated line in the background is the KSL).

**Figure 4:**
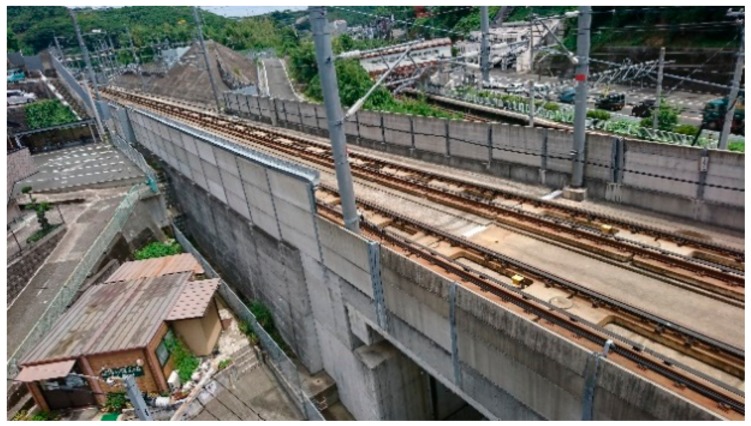
Shinkansen railway line. The forward barrier is 2 m high and the back barrier is 3 m high on the left-hand side.

The CRL with a 1.5 m high noise barrier (measured from the railway floor) was elevated in 2016 in the north area as shown in Figure 3. It has been elevated from Kumamoto station to a point 650 m south from the new station since 2010 and has been at ground level in the area located further south in the south area. The KSL has a noise barrier located 2–3 m above the railway floor in the survey area (Figure 4). No special noise measures have been taken for the KSL and CRL except for the elevation in the north area and the usual railway maintenance. Oerti emphasized that rolling stock improvement was preferable to noise barriers from the viewpoint of cost-effectiveness [16]. During the survey period, no substantial rolling stock improvement (such as introducing a new type of train) took place, and thus, the noise reductions might be attributed to the interventions alone.

The average *L*_Vmax_ and standard deviation are also shown in Table 4. The average *L*_Vmax_ of the Shinkansen railway decreased after the interventions. The reduction in the south area may be caused by the reduction in speed of the Shinkansen trains after the earthquakes. The *L*_Vmax_ of the conventional railway increased by 8 dB from 2012 to 2016 in the north area and decreased by 9 dB from 2011 to 2017 in the south area. The speed of conventional passenger cars slowed down because of the opening of the new station, and thus, *L*_Vmax_ decreased in the south area. On the other hand, apartment houses close to the railway track were occasionally included in the north area. The distribution of *L*_Vmax_ for the conventional railway in the north area is bimodal. The upper group refers to all apartment houses, and the lower, detached houses.

### 3.3. Evaluation of Environmental Factors after the Operation of the KSL in Comparison with the Conditions before the Operation

Railway noise, railway vibration, sunlight entering the respondents’ houses, and the view from their houses after the operation of the KSL were evaluated in comparison with the conditions before the operation of the KSL based on the following categories: (1) “better than before”; (2) “somewhat better”; (3) “neither better nor worse”; (4) “somewhat worse”; and (5) “worse”. Table 5 compares the rate of evaluations between the north area in 2016 and the south area in 2017. With regard to vibroacoustic factors such as noise and vibration, a higher number of “better” evaluations were recorded in the north area than in the south area, and a higher number of “worse” evaluations were noted in the south area than in the north area. As for visual factors such as the sunlight and view from the house, while the number of “better” evaluations was higher in the north area than in the south, there was little difference in the number of “worse” evaluations between both areas. Because the “better” evaluations were most consistent with regard to the vibroacoustic and visual factors and between the north and south areas, a Pearson’s *χ*^2^ test was applied to assess the independence of the environmental evaluations between the areas by classifying the evaluation categories into groups of the first two and the final three categories. There were significant differences for railway noise (*χ*^2^ = 112.43, *p* < 0.001), railway vibration (*χ*^2^ = 92.33, *p* < 0.001), sunlight (*χ*^2^ = 16.02, *p* < 0.001), and view (*χ*^2^ = 17.52, *p* < 0.001) between the north and south areas. It was found that the respondents in the north area perceived the situation regarding noise and vibration after the operation of the KSL to be much better compared with the respondents in the south area.

Furthermore, 26 and 51 free opinions were obtained regarding the living environments in the north and south areas, respectively. Four positive opinions (i.e., no signal sound, and decrease in noise and vibration due to the elevation) and three negative opinions were obtained regarding railway noise and vibration in the north area. Four positive opinions (i.e., used to the noise and vibration) and eight negative opinions (i.e., more annoyed by noise and vibration after the earthquakes) were obtained in the south area. These findings are consistent with the information in Table 5, implying that the respondents in the south area were more concerned about environmental problems because of their perception of poor environmental improvement. Thus, respondents in the south area were found to be more annoyed than those in the north area by railway noise and vibration.

### 3.4. Cross Tabulation of Frequency of High Annoyance between L_den_ and before/after the Earthquakes

The cross–tabulated responses of highly annoyed respondents between *L*_den_ and before/after the earthquakes for the detached and apartment houses in the north and south areas are shown in Table 6. The total frequency of high annoyance is reasonably small for residents of detached houses in the north area, i.e., 8 and 4 before and after the earthquakes, respectively. These sample sizes are too small for an analysis using high annoyance as a dependent variable in multivariate analyses. Therefore, it was reasonable to conduct further analysis using data from the south area only. The mode of “highly annoyed” responses for both detached and apartment houses are in the range from 53 to 62 dB in terms of *L*_den_ before the earthquakes, but the range decreased to 48–52 dB after the earthquakes.

### 3.5. Evaluation of the Residential Environment of the South Area in 2011 and 2017

Since surveys for non-respondents were not carried out and the response rates of the 2011 and 2017 surveys were quite low (as shown in Table 2), the representativeness of the population is not guaranteed. However, to validate the homogeneity of the respondents before and after the earthquakes, the general evaluations of residential environments are compared for 2011 and 2017. Nine items in the residential environments were evaluated in the questionnaire: green area, townscape, view from the residents’ houses, quietness around their houses, and convenient access to workplaces, schools, health care facilities, shopping, and transportation. The former three factors were selected for the comparison between 2011 and 2017 because the latter six could be affected by the interventions. These items were evaluated using a 5-point verbal scale (“extremely good,” “good,” “neither good nor bad”, “bad” and “extremely bad”), and the relative frequency of responses is shown in Table 7. To test the independence between 2011 and 2017, Pearson’s *χ*^2^ test was applied and the results showed no statistical difference (green area: *χ*^2^ = 2.46, *p* = 0.651; townscape: *χ*^2^ = 5.755, *p* = 0.218; and view from residents’ houses: *χ*^2^ = 2.91, *p* = 0.574). This analysis confirmed that the respondents’ attitudes to non-acoustic factors, as shown in Table 7, remained the same before and after the earthquakes. This result suggests that the respondents were homogenous before and after the earthquakes in the south area.

### 3.6. Exposure–Annoyance Relationships in the South Area

**Figure 5:**
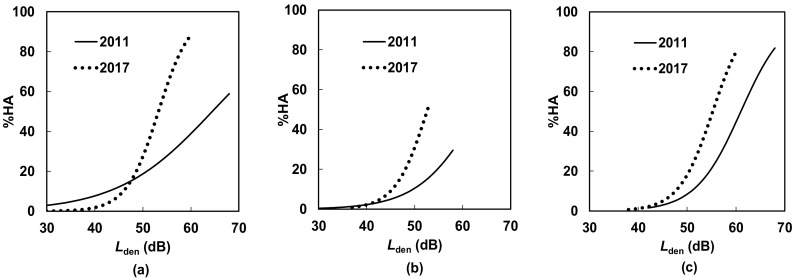
Comparison of exposure–response relationships for detached house residents before and after the opening of the new station in the south area: (**a**) conventional railway noise; (**b**) Shinkansen railway noise; and (**c**) total railway noise.

**Figure 6:**
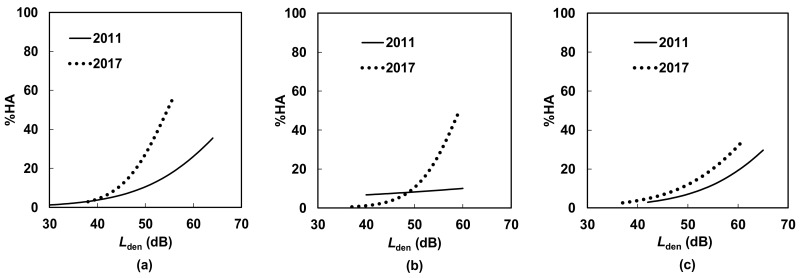
Comparison of exposure–response relationships for apartment house residents before and after the opening of the new station in the south area: (**a**) conventional railway noise; (**b**) Shinkansen railway noise; and (**c**) total railway noise.

Figure 5 and Figure 6 compare the *L*_den_–%Highly Annoyed (%HA) relationships for conventional railway, Shinkansen, and total railway noise in the south area before and after the operation of the new station for detached house and apartment house residents, respectively. As noise and vibration propagation differ between the detached and apartment houses, the exposure–annoyance relationships are described separately. In Figure 5, although *L*_den_ decreased from 2011 to 2017, noise annoyance increased for both conventional and Shinkansen railways, but was higher for conventional railway noise in particular. A similar but slightly more moderate trend can be found in Figure 6.

### 3.7. Multiple Logistic Regression Analysis

To elucidate factors affecting high annoyance in detail, a multiple logistic regression analysis was applied. The results of the multiple logistic regression analysis of high annoyance due to total railway noise for detached house and apartment house residents are shown in Table 8 and Table 9, respectively. The Areas Under the Curve (AUCs) for high annoyance expressed by the detached and apartment house residents were 0.79 and 0.76, respectively. These results indicate that the predictive abilities of the models are moderately accurate. The results pertaining to noise from the conventional and Shinkansen railways showed similar trends (not shown here). For detached house residents, *L*_den_, sensitivity to noise, and serious damage by earthquakes significantly affected high annoyance, whereas sex, age, use of trains, and the frequency of opening living room windows did not significantly affect annoyance. For apartment house residents, the findings were similar, except the frequency of opening windows significantly affected annoyance. As apartment houses are directly exposed to noise, these residents are apt to be affected more by opening windows than residents of detached houses. Notably, van Gerven et al. reported a curvilinear relationship showing the maximum value for respondents in the 40s [17], but no evidence of the same was found in this study.

## 4. Discussion

The most important findings in this study are that minimal damage caused by the earthquakes did not significantly affect the level of annoyance of the residents of detached and apartment houses, whereas serious damage significantly affected the annoyance of the residents of both house types in the south area. Specifically, the former finding shows that minimal earthquake damage in addition to a step change in railway noise exposure did not yield significant change in annoyance, whereas serious earthquake damage in addition to a step change in noise exposure resulted in significant change in annoyance. However, because the odds ratio of “minimal damage” relative to “no damage” was 1.99 for detached house residents and 2.85 for apartment house residents, even minimal damage by earthquakes did appear to increase annoyance. This shows that there was an “under response” in the south area because annoyance increased while noise exposure decreased. Comparison of Figure 5 and Figure 6 and of Table 8 and Table 9 showed that the effect of earthquake damage for detached house residents appeared slightly greater than that for apartment house residents. Unfortunately, the effects of the operation of the new station alone on railway noise annoyance could not be investigated in this study.

It is important to consider why the earthquakes affected annoyance. We hypothesize as follows. After the earthquakes, aftershocks accompanied by loud rumbling sounds from the ground occurred frequently for some time. Such a situation possibly made people so sensitive to vibration as to detect even small vibrations. They could respond sensitively to vibrations other than aftershocks, such as those due to the railways. As a result, they started fearing intermittent railway vibrations, leading to increased vibration annoyance. Since railway vibrations, which are usually accompanied by noise, affect people negatively, noise annoyance could have increased. Thus, earthquakes are considered to be a unique intervention in this context. Peris et al. investigated the effect of situational, attitudinal, and demographic variables on railway vibration annoyance and found that the annoyance was strongly affected by concerns about property damage and expectations about future vibration levels [18]. The former finding is consistent with and develops our hypothesis: concerns about property damage by earthquakes could enhance concerns about property damage by railway vibration only, thus increasing noise annoyance. A similar trend has also been found in aircraft noise studies, where the fear of an aircraft crash significantly increased the level of noise annoyance around airports [19]. However, this hypothesis on the mechanism between earthquakes and noise annoyance should be validated with further research, since this phenomenon was newly observed in this study. The latter finding on the expectation about future levels of vibration also requires further research in terms of how long these effects (caused by earthquakes) might continue.

Although the results for the north area are not shown in this paper because of the fewer responses expressing high levels of annoyance, it could be useful to investigate the differences in environmental perception between the residents in the north and south areas to interpret the reasons for the lower level of annoyance in the north area. As shown in Table 5, the residential environment after the operation of the KSL, particularly in terms of noise and vibration, was perceived to be much better by respondents in the north area than in the south, although the levels of noise exposure were similarly reduced in both areas (see Table 4). In the north area, all respondents were able to visually and aurally to perceive the change in their environments following the construction of the elevated railway. Consequently, the scenery along the railway in the north area changed markedly. In the south area, the respondents might not have perceived a change in the environment visually, except for those living close to the new station. We hypothesize that such a difference in perception of environmental change could account for the difference in the overall impression of environmental improvement between the north and south areas.

This hypothesis might be enhanced by the findings in the previous studies on the visual effect on noise annoyance [20,21,22,23,24]. Joynt and Kang reported that the perception of noise from roads located behind the barriers was affected by the preconception that the barrier material would reduce noise, and thus, the aesthetics of the barriers and noise perception were inversely correlated [20]. Maffei et al. indicated that the perception of railway noise was judged to be lower for transparent barriers than opaque ones and that this difference increased as noise level increased [21]. Van Renterghem and Botteldooren revealed that the amount of vegetation viewed through the living room window facing a city road significantly reduced the self-reported noise annoyance [22]. Pedersen and Larsman showed that wind turbine noise (WTN) annoyance was higher when the wind turbine was visible [23]. Aletta et al. investigated the effect of vision on perception caused by a chiller and showed that while the distance between the chiller and the respondents influenced perception, the visibility of the chiller did not [24]. Aletta et al. also tried to explain the difference in the main findings from the abovementioned studies for different sound levels and the spectral and temporal characteristics of the sound sources: the visibility increased the perception for road traffic noise with relatively high sound levels [20] while it decreased the perception for railway noise with similar sound levels [21]. They speculated that this may be because people might prefer to see the source when the sound level is not as constant as that for road traffic. The difference between the results for WTN and chiller noise was attributed to specific spectral and temporal characteristics such as low frequency components and amplitude modulation for WTN, and the difference in context such as rural background for WTN and peri-urban for chiller noise [23,24].

The visibility of the noise source and vegetation and the material of the noise barrier are important factors in railway noise annoyance research, as shown in previous studies. However, this study did not compare the annoyance response between areas; rather it conducted the comparison between before and after the interventions. The noise barriers in the survey area are made of precast reinforced concrete as shown in Figure 3 and Figure 4. The living rooms of the surveyed houses principally face south (they do not directly face the railway), and the residents can see their gardens or neighboring houses through their living room windows. The amount of vegetation that the residents could see through their living room windows was not considered to be largely different between before and after the earthquake. Table 7 also shows practically no difference in distribution of visual responses to the residential area between before and after the earthquakes. Hence, the visual effect of noise source, noise barriers, and vegetation on noise annoyance was not essentially different between before and after the earthquakes.

To the best of our knowledge, other than Nagahata et al. [2,3], no study has explored the effects of earthquakes on noise annoyance. Nagahata et al. suggest that when a serious earthquake occurs in an area, special care should be given to the victims. In addition to the aftershocks, the respondents were found to be highly stressed by the application process for support from the local government to repair their houses. Therefore, the government should urgently increase efforts to reduce such stress. Furthermore, it takes considerable time to restore infrastructure damaged by earthquakes. However, the precise roadmap for restoration should be made available to local residents as quickly as possible.

Regarding noise policies, providing the local population with prior information regarding planned interventions is essential, as highlighted by Schreckenberg [9,11] and Lam [12]. Currently, there appears to be no active effort to provide information to local communities regarding the expected effects of the railway elevation and the operation of the new station. Either the government or the railway company should make it known that the railway elevation and the operation of the new station will decrease noise and vibration.

This study was originally planned to investigate the effects of railway elevation and the operation of a new station on the local population. However, two large earthquakes struck the Kumamoto region unexpectedly just after these interventions. Thus, this study transformed into a special case study showing the effect of big earthquakes on noise annoyance. This research is notable as such a condition seldom occurs.

## 5. Conclusions

This study compared the level of annoyance caused by railway noise before and after the interventions of the elevation of the railway (Type 2) in the north area, the operation of the new station (Type 1) in the south area, and the occurrence of the earthquakes. However, only the effects of the operation of the new station and the occurrence of the earthquakes in the south area were discussed because of the few expressions of high levels of annoyance in the north area. Although the operation of the new station decreased conventional railway noise exposure considerably, the effects of only the operation of the new station on railway noise annoyance could not be analyzed. However, minimal damage caused by the earthquakes in addition to the intervention increased annoyance moderately, whereas serious damage caused by the earthquakes in addition to the intervention increased annoyance significantly. This finding strongly suggests that earthquakes increase noise annoyance substantially.

## Figures and Tables

**Table 1:** Social survey plan and events.

Date	Area	Railway Situation	Survey
August–September 2009	North	Conventional 1st temporary line	I
July–August 2010	South	Conventional line	
12 March 2011		Opening of the Kyushu Shinkansen Line	
April–May 2011	North	Shinkansen + Conventional 1st temporary line	II
August–September 2011	South	Shinkansen + Conventional line	
July–August 2012	North	Shinkansen + Conventional 2nd temporary line	III
14–16 April 2016		Kumamoto earthquakes	
November–December 2016	North	Shinkansen + Conventional elevated line	IV
July–September 2017	South	Shinkansen + Conventional line with new station	V

**Table 2:** Number of respondents, response rate, and distribution of demographic variables.

Survey	III (2012)			IV (2016)			II (2011)			V (2017)		
House type	D *^1^	A *^2^	Total	D	A	Total	D	A	Total	D	A	Total
No. of deliveries	312	787	1099	456	708	1164	612	710	1322	758	498	1256
No. of responses	143	190	333	279	120	399	236	139	375	253	75	328
Response rate (%)	45.8	24.1	30.3	61.2	16.9	34.2	38.6	19.6	28.4	33.4	15.1	26.1
Sex (%)												
Male	46.5	40.0	42.8	44.7	44.1	44.5	37.5	33.8	36.1	51.4	25.7	45.5
Female	53.5	60.0	57.2	55.3	55.9	55.5	62.5	66.2	63.9	48.6	74.3	54.5
Age (%)												
<30	4.3	28.4	18.2	0.7	16.0	5.3	6.1	8.7	7.0	3.2	9.5	4.6
30s	3.6	12.6	8.8	3.3	14.3	6.6	11.3	13.8	12.2	6.8	24.3	10.8
40s	13.6	15.8	14.9	9.5	10.1	9.6	20.4	12.3	17.3	13.7	18.9	14.9
50s	14.3	18.4	16.7	13.5	16.8	14.5	13.9	20.3	16.3	16.1	20.3	17.0
60s	28.6	12.6	19.4	31.6	26.1	30.0	23.8	21.0	22.8	22.9	14.9	21.1
≥70	35.7	12.1	22.1	41.5	16.8	34.0	24.7	23.9	24.4	37.4	12.2	31.6
No. of family members (%)												
1	23.6	51.1	39.3	21.3	35.6	25.6	17.5	22.1	19.2	16.1	21.6	17.3
2	30.0	18.1	23.2	37.5	36.4	37.2	33.8	29.4	32.1	34.5	40.5	35.9
3	24.3	13.3	18.0	21.7	13.6	19.2	18.4	26.5	21.4	21.3	21.6	21.4
4	15.0	14.9	14.9	9.9	11.0	10.3	15.4	16.9	15.9	14.9	12.2	14.2
5	5.0	2.1	3.4	5.5	3.4	4.9	8.8	3.7	6.9	9.6	4.1	8.4
6	2.1	0.5	1.2	3.3	-	2.3	4.4	1.5	3.3	2.8	-	2.2
7	-	-	-	0.7	-	0.5	0.9	-	0.5	0.8	-	0.6
8	-	-	-	-	-	-	0.9	-	0.5	-	-	-
Owned (%)	84	32	54	93	46	79	75	70	73	92	18	75

*^1^: Detached; *^2^: Apartment.

**Table 3:** Number of passing trains during daytime, evening, and nighttime.

Survey	Period	Local		Shinkansen		Freight		Total	
		III	IV	III	IV	III	IV	III	IV
North area	Daytime (07:00–19:00)	56	56	98	87	4	2	158	145
	Evening (19:00–22:00)	13	12	24	24	3	3	40	39
	Nighttime (22:00–7:00)	15	14	13	16	5	7	33	37
	Total	84	82	135	127	12	12	231	221
**Survey**	**Period**	**II**	**V**	**II**	**V**	**II**	**V**	**II**	**V**
South area	Daytime (07:00–19:00)	94	101	71	84	4	2	169	187
	Evening (19:00–22:00)	24	24	21	17	2	3	47	44
	Nighttime (22:00–7:00)	23	22	30	29	6	5	59	56
	Total	141	147	122	130	12	10	275	287

**Table 4:** Averages and standard deviations of noise exposure.

Source	Metric	Survey III		Survey IV		Survey II		Survey V	
		2012 (North)		2016 (North)		2011 (South)		2017 (South)	
		Mean	S.D.	Mean	S.D.	Mean	S.D.	Mean	S.D.
Conventional railway	*L*_Aeq,24h_	45	11.4	37	6.8	43	9.5	39	5.8
	*L*_Aeq,d_	46	11.4	38	6.7	44	9.5	41	5.9
	*L*_Aeq,e_	45	11.5	36	7.2	44	9.5	39	5.7
	*L*_Aeq,n_	41	11.3	33	6.8	41	9.5	34	5.5
	*L*_den_	49	11.4	41	6.8	49	9.5	43	5.7
	*L*_Vmax_	40	4.4	48	8.4	51	5.2	42	14.5
Shinkansen	*L*_Aeq,24h_	46	7.1	43	4.9	41	5.1	39	3.3
	*L*_Aeq,d_	48	7.1	45	4.9	41	5.1	41	3.4
	*L*_Aeq,e_	46	7.4	45	4.9	42	5.2	39	3.2
	*L*_Aeq,n_	38	7.2	39	4.9	38	5.2	37	3.3
	*L*_den_	48	7.2	47	4.9	46	5.1	44	3.3
	*L*_Vmax_	46	3.0	45	1.1	46	2.5	40	6.2
Total railway	*L*_Aeq,24h_	50	8.3	45	4.9	47	5.0	43	3.8
	*L*_Aeq,d_	51	8.2	46	4.9	48	5.0	44	3.9
	*L*_Aeq,e_	50	8.4	46	4.8	48	5.0	43	3.8
	*L*_Aeq,n_	44	9.1	40	5.0	45	5.2	39	3.7
	*L*_den_	53	8.6	49	4.9	52	5.1	47	3.7

**Table 5:** Evaluation of environmental factors compared with before the start of the KSL operation (%).

Category	Railway Noise		Railway Vibration		Sunlight		View from House	
	2016 North	2017 South	2016 North	2017 South	2016 North	2017 South	2016 North	2017 South
Better than before	43	9	42	8	17	7	16	7
Somewhat better	19	12	16	12	6	4	8	4
Neither better nor worse	32	63	40	67	67	79	44	57
Somewhat worse	4	14	1	10	6	7	20	20
Worse than before	2	2	1	2	4	2	13	12

**Table 6:** Cross-tabulation of “highly annoyed” responses for *L*_den_ and before/after the earthquakes for detached and apartment houses in the north and south areas.

*L*_den_	North Area				South Area			
	Detached		Apartment		Detached		Apartment	
	Before	After	Before	After	Before	After	Before	After
23–27	1	-	-	-				
28–32	0	0	-	-				
33–37	0	0	1	-	-	-	-	0
38–42	1	0	0	-	-	2	0	-
43–47	0	1	0	1	4	4	0	0
48–52	0	3	1	2	5	16	3	6
53–57	0	0	1	0	11	3	7	2
58–62	4	-	3	5	11	1	6	1
63–67	2	-	11	2	1	-	1	-
68–72	1	-	0	-	0	-	-	-
Total	8	4	17	10	32	26	17	9

**Table 7:** Relative frequency of evaluation of the residential environment of the south area in 2011 and 2017 (%).

Result	Green Area		Townscape		View from Houses	
	2011	2017	2011	2017	2011	2017
Extremely good	8.2	8.0	5.0	1.9	6.3	6.6
Good	35.5	35.7	20.3	22.5	19.7	21.9
Neither good nor bad	48.4	45.1	58.1	57.5	51.9	47.5
Bad	7.2	10.4	15.3	16.2	20.9	21.3
Extremely bad	0.6	0.8	1.3	1.9	1.3	2.7

**Table 8:** Multiple logistic regression analysis of high annoyance toward total railway noise for detached house residents in the south area (AUC = 0.79).

Item	Category	Estimate	Standard Error	*p* Value	Odds Ratio	Lower 95% CI	Upper 95% CI
Intercept		−15.461	2.449	<0.001			
*L*_den_^a^		0.249	0.043	<0.001	1.283	1.184	1.402
Sex	Male				1		
	Female	0.148	0.336	0.658	1.160	0.602	2.257
Age	<40				1		
	40 s	−0.517	0.757	0.495	0.597	0.126	2.641
	50 s	−0.254	0.663	0.701	0.776	0.213	2.999
	60 s	0.496	0.610	0.416	1.642	0.522	5.900
	≥70 s	0.538	0.610	0.378	1.713	0.545	6.169
Sensitivity	Not sensitive				1		
	Sensitive	1.020	0.338	0.003	2.774	1.427	5.395
Use of trains	Use				1		
	Not use	0.028	0.341	0.935	1.028	0.521	1.998
Frequency of opening windows	Close				1		
	Open	−0.408	0.351	0.246	0.665	0.332	1.324
Damage of earthquake	No (2012)				1		
	Minimal (2016)	0.688	0.528	0.193	1.990	0.687	5.567
	Serious (2016)	1.232	0.460	0.007	3.427	1.404	8.599

^a^: Odds ratio for a change of 1 dB in the noise level.

**Table 9:** Multiple logistic regression analysis of high annoyance toward total railway noise for apartment house residents in the south area (AUC = 0.76).

Item	Category	Estimate	Standard Error	*p* Value	Odds Ratio	Lower 95% CI	Upper 95% CI
Intercept		−10.240	3.231	0.002			
*L*_den_^a^		0.133	0.056	0.017	1.142	1.029	1.282
Sex	Male				1		
	Female	0.135	0.511	0.792	1.144	0.432	3.271
Age	<40				1		
	40s	−0.365	0.634	0.565	0.694	0.186	2.323
	50s	−0.829	0.743	0.264	0.436	0.085	1.705
	60s	−0.251	0.672	0.709	0.778	0.193	2.802
	≥70s	−1.264	0.943	0.180	0.283	0.033	1.525
Sensitivity	Not sensitive				1		
	Sensitive	1.378	0.485	0.005	3.967	1.545	10.524
Use of trains	Use				1		
	Not use	−0.272	0.482	0.573	0.762	0.287	1.934
Frequency of opening windows	Close				1		
	Open	1.218	0.538	0.024	3.379	1.220	10.270
Damage of earthquake	No (2012)				1		
	Minimal (2016)	1.047	0.837	0.211	2.850	0.497	14.498
	Serious (2016)	1.673	0.675	0.013	5.328	1.396	20.537

^a^: Odds ratio in 1 dB change.

## Appendix A

Questions about noise annoyance (responses collected in terms of an 11-point numerical scale):

“Thinking about the last 12 months or so, what number from 0 to 10 best shows how much you are bothered, disturbed, or annoyed at home by the following noise?”

(1) Conventional railway noise
     0  1  2  3  4  5  6  7  8  9  10
  Not at all                        Extremely
(2) Shinkansen railway noise
     0  1  2  3  4  5  6  7  8  9  10
  Not at all                        Extremely
(3) Total of conventional and Shinkansen railway noise
     0  1  2  3  4  5  6  7  8  9  10
  Not at all                        Extremely

The above questions were used in Surveys III–V but the phrase “… the last 1 month or so …” was used in Survey II.

## Appendix B

According to JIS C 1510, the measured vibration level *L*_v_ is defined as follows:

$$L_{v}=10\log\left(\sum \frac{a_{n}{}^{2}10^{\frac{c_{n}}{10}}}{a_{0}{}^{2}}\right)\;\left[\mathrm{dB}\right]$$

- *a_n_*: acceleration (m/s^2^)
- *c_n_*: relative response (see Table A1)
- *a*_0_: reference acceleration, 10^−5^ m/s^2^

**Table A1:** Frequency weighting for vertical vibration (JIS C 1510).

Frequency (Hz)	1	2	4	6.3	8	16	32	63	80
Cn (dB)	−5.9	−3.2	0.1	0.2	−0.9	−6.1	−12.0	−18.0	−20.0
